# Highly Efficient Recovery and Recycling of Cobalt from Spent Lithium-Ion Batteries Using an *N*-Methylurea–Acetamide Nonionic Deep Eutectic Solvent

**DOI:** 10.1021/acsomega.2c07780

**Published:** 2023-02-13

**Authors:** Subramanian Suriyanarayanan, Mohana Priya Babu, Raja Murugan, Divyamahalakshmi Muthuraj, Kothandaraman Ramanujam, Ian A. Nicholls

**Affiliations:** † Bioorganic and Biophysical Chemistry Laboratory, Linnaeus Centre for Biomaterials Chemistry, Department of Chemistry and Biomedical Sciences, Linnaeus University, SE-39182 Kalmar, Sweden; ‡ Clean Energy Laboratory, Department of Chemistry, Indian Institute of Technology Madras, Chennai, Tamil Nadu 600 036, India

## Abstract

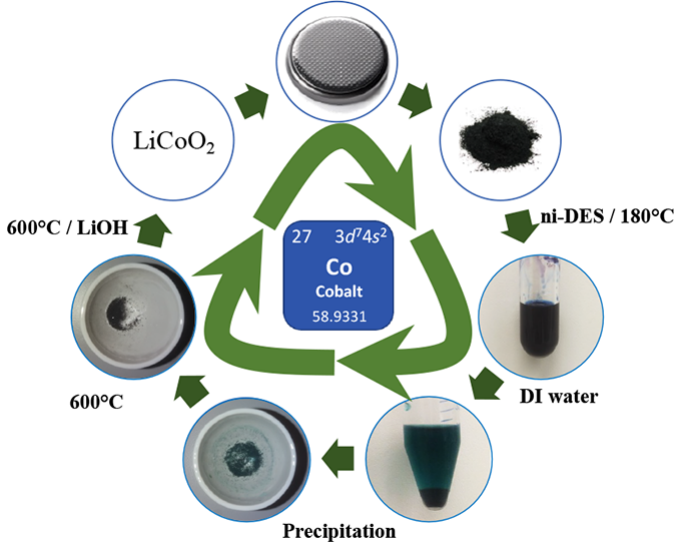

The growing demand for lithium-ion batteries (LiBs) for the electronic and automobile industries combined with the limited availability of key metal components, in particular cobalt, drives the need for efficient methods for the recovery and recycling of these materials from battery waste. Herein, we introduce a novel and efficient approach for the extraction of cobalt, and other metal components, from spent LiBs using a nonionic deep eutectic solvent (ni-DES) comprised of *N*-methylurea and acetamide under relatively mild conditions. Cobalt could be recovered from lithium cobalt oxide-based LiBs with an extraction efficiency of >97% and used to fabricate new batteries. The *N*-methylurea was found to act as both a solvent component and a reagent, the mechanism of which was elucidated.

## 1 Introduction

The dramatic rise in consumption of rechargeable lithium-ion batteries (LiBs) has been driven by the energy-dependent communications, electronics, and automobile industries.^1^ Both the number of electric vehicles (EVs) and the size of batteries are rapidly increasing, with EVs expected to account for nearly two-thirds of all cars sold worldwide by 2040.^2^ This shall in turn lead to significant amounts of LiB-derived waste.^3^ Currently, spent LiBs are most often stockpiled or discarded in landfills, which poses a serious threat to the environment^4^ and public health^5^ and accelerates the depletion of these important resources,^6^ if they are not recycled or reused. Cobalt is critical for the production of LiB cathode components, constituting up to 15 wt % of the cathodes.^7^ The gap between the supply and demand for cobalt is widening and is expected to increase by 16% a year through 2030.^8,9^ Accordingly, sustainable methods for recycling spent LiBs are desirable, as efficient processes for the recovery of critical LiB elements have yet to be developed.

The primary value present in spent LiBs lies in the metal oxides in the LiB cathode. Extraction of critical metals from the active lithium oxides layers is usually carried out either by pyrometallurgy,^10^ hydrometallurgy,^11^ biometallurgy,^12^ or a combination of these techniques.^12,13^ While pyrometallurgy is arguably state-of-the-art in this regard, it suffers from high energy costs, the need for extreme temperatures higher than 1400 °C,^14^ difficulties in the comprehensive recovery of metals, and the generation of harmful gases.^15^ Hydrometallurgy affords high extraction efficiencies,^16^ though it necessitates the use of extreme chemical conditions, e.g., alkali metal hydroxides and concentrated acids.^17^ Green solvents, such as ionic liquids^18,19^ and organic acids,^20^ generally require additional reagents to accelerate the extraction process, and their stability, production cost, and waste disposal are problematic.^21^

**Scheme 1:**
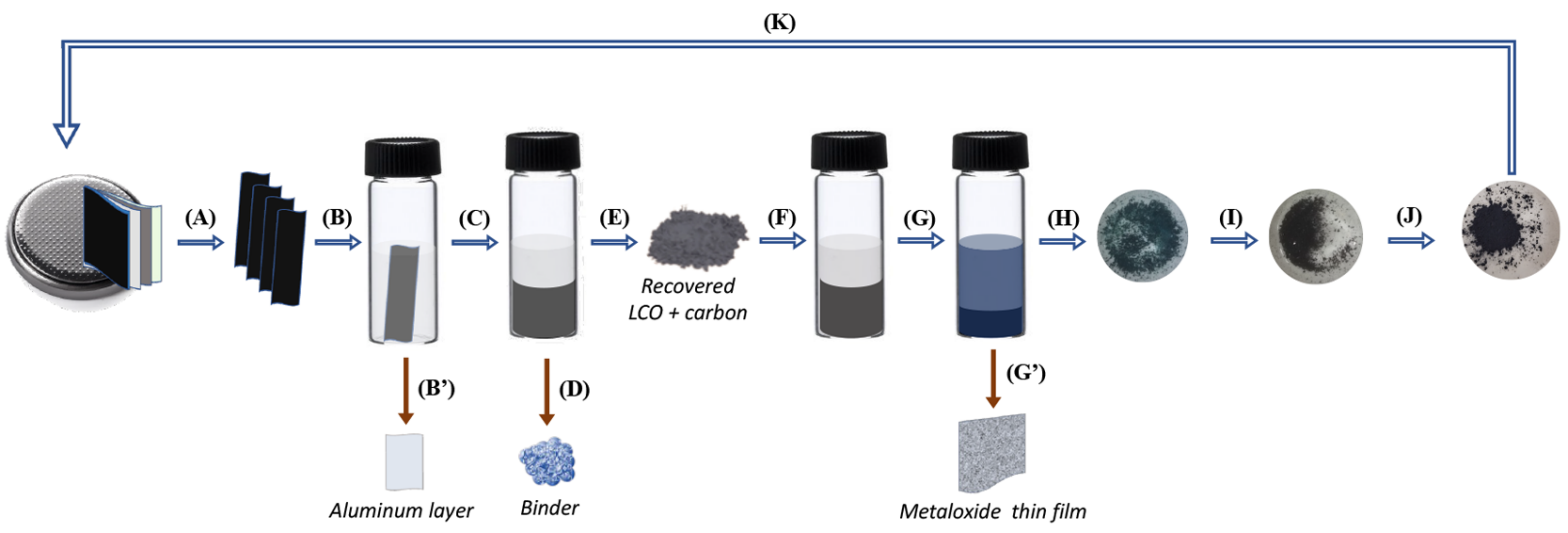
Schematic Representation Denoting the Recovery and Recycling of Cobalt from the Cathode Materials of Spent LiBs (A) Dismantlement of discharged LiB to separate cathode layers; (B) sonication of cathode layers in *N*-methyl-2-pyrrolidone (NMP); (B′) separation of the aluminum layer; (C) centrifugation; (D) removal of supernatant NMP containing the PvDF binder; (E) drying of the pelleted cathode material (LCO) in a hot air oven at 120 °C for 2 h; (F) dispersion of collected LCO in NMU-A ni-DES; (G) heat treatment of collected LCO with NMU-A ni-DES at 180 °C for 24 h; (G′) dissolution of the leachate in 1% acetic acid, followed by the addition of KNO_3_ (0.1 M) and the electrodeposition of cobalt hydroxide (see section 4.2.9); (H) dissolution of the leachate in Milli-Q grade water, followed by centrifugation to recover the cobaltous precipitate and drying in a hot air oven at 120 °C for 12 h; (I) calcination of the recovered cobaltous powder at 500 °C for 5 h; (J) addition of the required amount of lithium hydroxide, followed by calcination at 500 °C for 5 h; and (K) fabrication of a new LiB (see section 4.2.10).

In the present work, we demonstrate the use of recently described amide-based nonionic deep eutectic solvents (ni-DESs)^22^ for the highly efficient extraction and recovery of cobalt from spent LiBs without the need for additional reagents. For that, we have used lithium cobalt oxide (LiCoO_2_, LCO), as a model compound, which has generally been used as cathode material in LiBs batteries. Metals recovered from LCO by ni-DES extraction were converted to cobalt oxides (Co_2_O_3_) in the particulate form and thereafter used to produce new thin-film cathodes for fabricating functional LCO-LiBs (Scheme 1). Alternatively, the thin-film formats of cobalt hydroxides (Co(OH)_2_) can also be prepared by electrodeposition methods and can be used as heterogeneous catalysts.

Novel ni-DESs derived from mixtures of urea and acetamide derivatives have been shown to be helpful for solubilizing a wide range of organic and inorganic species.^22^ These nonionic deep eutectic solvents were developed and studied with respect to composition and the mechanisms underlying their properties in a previously published study presented by us. The phase diagrams constructed with urea and acetamide derivatives show the eutectic temperature and the corresponding composition.^22^ These novel solvents have been prepared with readily available and biodegradable compounds classified as nonhazardous and noncarcinogenic (US-EPA and ECHA) for humans.^23^ The utility of this family of ni-DESs has even been demonstrated in polymer synthesis,^24^ organic synthesis, and natural product extraction.^25^ Furthermore, the ni-DESs are highly soluble in water and can be recovered and recycled. The unique solvation properties of these ni-DESs could be exploited for the recovery of critical metals, e.g., cobalt and lithium, from LiB waste for use in new-battery fabrication (Scheme 1).

## 2 Results and Discussion

**Figure 1:**
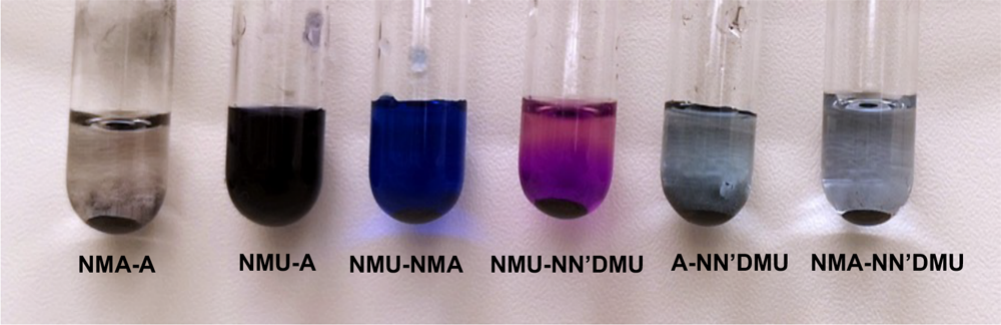
Extraction of cobalt from LiCoO_2_ using various ni-DESs comprising acetamide and urea derivatives. Briefly, 20 mg of the LCO powder was dispersed in 2 mL of the ni-DES, and the mixture was heated at 180 °C for 24 h (photograph taken directly after removal from the heating source).

To identify candidate ni-DES systems to establish the proof-of-concept, a series of six ni-DESs^22^ (Table S1) were prepared from urea and acetamide derivatives, and their capacities to extract cobalt from LCO while heating at 180 °C for 24 h were determined (Figure 1). An intense dark blue color developed in *N*-methylurea (NMU)-containing ni-DESs indicates the prompt extraction of cobalt from LCO. The ni-DES comprised of *N*-methylurea (NMU) and acetamide (A) (50:50, w/w) was found to extract cobalt most efficiently from the LCO powder under these conditions, as could be observed through the intense color of the extract and quantified by ICP-AES analysis (Table 1).

**Table 1:** Extraction of Metals from LiCoO_2_ (LCO) using different ni-DESs^a^

extracted metal	ni-DES	average concentration *c* (ppm) × 10^2^	standard deviation ×10^2^	extraction efficiency η (%)
Co	NMA-A	0.11	0.011	0.18
	NMU-A	54	3.90	91
	NMU-NMA	45	2.6	75
	NMU-NN’DMU	34	2.1	57
	A-NN’DMU	10	1.1	17
	NMA-NN’DMU	9.7	1.0	16
Li	NMA-A	0.0014	0.0012	0.02
	NMU-A	6.4	1.0	91
	NMU-NMA	5.1	0.68	73
	NMU-NN’DMU	3.9	0.58	56
	A-NN’DMU	0.71	0.12	10
	NMA-NN’DMU	0.79	0.15	11

a 20 mg of LCO powder was initially added and mixed with a finite amount (2 mL) of ni-DES, and the mixture was heated at 180 °C. Metal concentrations were averaged, and the standard deviations are for three different measurements at each temperature. All the values are standardized for two significant figures.

**Figure 2:**
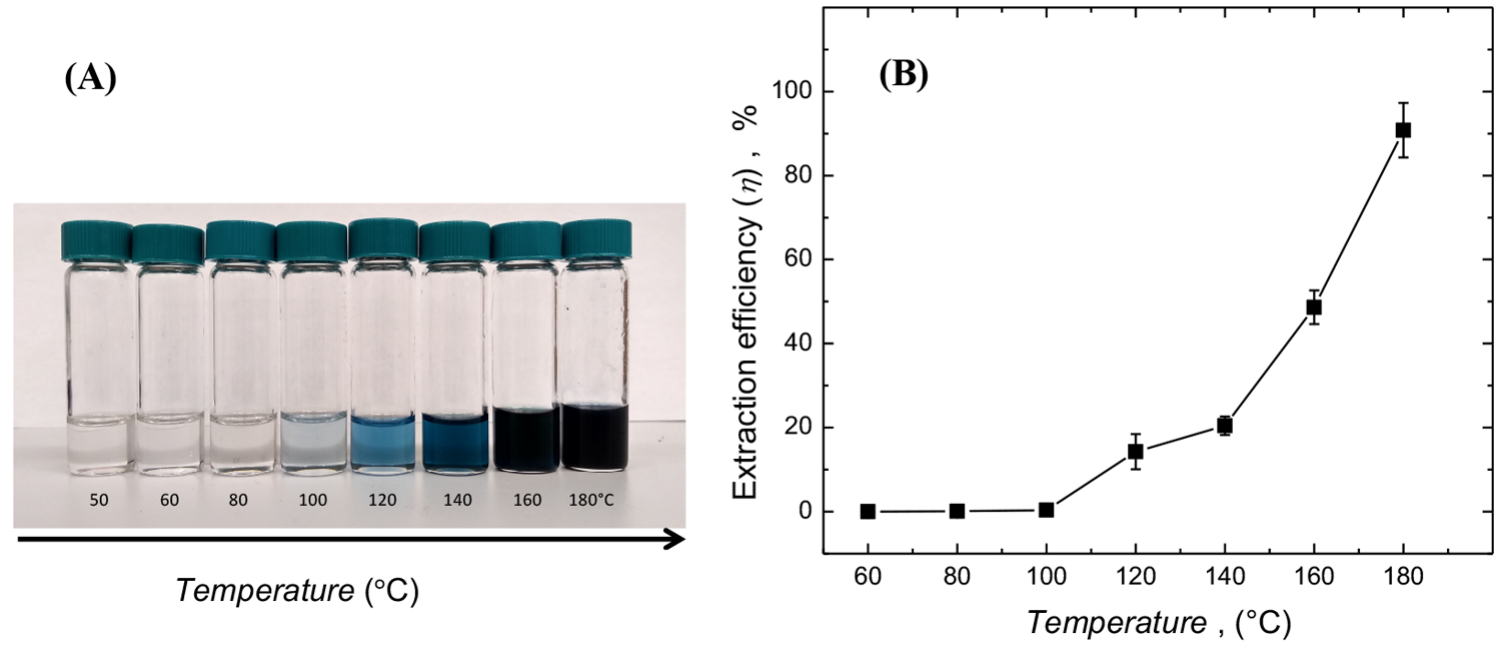
(A) Color change observed upon heating LiCoO_2_ in NMU-A ni-DES at 50–180 °C (±3 °C) for 24 h. (B) Extraction efficiency of cobalt as determined by the ICP-AES measurement upon heating LiCoO_2_ at different temperatures in NMU-A ni-DES for 24 h.

For the conservation of energy, lower temperatures and extraction times are desirable. In a study over 50–180 °C, ICP-AES analysis revealed a dramatic increase in efficiency above 140 °C (Figure 2 and Table S2), with a maximum extraction efficiency (90.78%) at 180 °C. When extraction times were varied from 12 to 48 h (Table S3), some further improvement, up to 97.66%, was obtained using 48 h extraction. This trend in the extraction efficiency is directly comparable to the reported efficiencies of the hydrometallurgical extraction process using caustic reagents, such as phosphoric acid (97.8%)^26^ and concentrated hydrochloric acid (100%),^27^ for the extraction of cobalt from *e*-waste. The extraction efficiency for lithium (Table S2) is also comparable to that of the cobalt, indicating the nonselective nature of leaching and subsequent extraction process using ni-DES. This observation shall be discussed again later. An NMU-A (2 mL) extraction of 100 mg of LCO at 180 °C for 24 h recovered ≈82% of cobalt (≈ 49 mg) (Table S4). In the ideal situation, this would correspond to ≈0.4 L of solvent for ≈82% cobalt recovery from a typical smartphone, which contains an average of 8–10 g of cobalt.^28^ The solid-to-liquid mass ratio is comparable to the state-of-the-art hydrometallurgical cobalt extraction from LiBs.^29^

**Figure 3:**
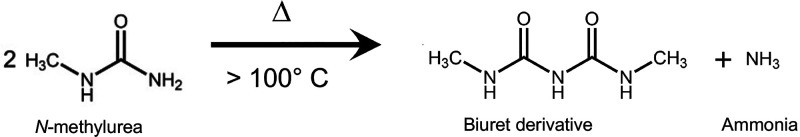
Self-reaction of NMU when heated above 100 °C.

The nonlinear temperature dependence of the extraction efficiency prompted an investigation of the mechanism of the NMU-A extraction of cobalt from LCO. A series of UV–vis and FT-IR spectroscopic studies revealed that at temperatures above 100 °C, NMU can undergo a condensation reaction to produce a biuret derivative and ammonia (Figure 3),^30^ analogous to that of urea. Therefore, both the biuret product and ammonia were anticipated to be able to interact with cobalt,^31^ thus facilitating its extraction from the temperature-aged LiCoO2.

The characteristic blue color of the extract can be attributed to the complexation of Co^3+^, as reflected in the UV–vis spectral absorption at ∼560 nm from the ni-DES containing cobalt extracted at 120 °C (Figure S1-A). For extraction temperatures above 120 °C, a red shift was observed at higher temperatures, for example, 584 nm for extraction at 180 °C. This shift was attributed to the increased degree of complexation of cobalt ions by a greater concentration of biuret and ammonia. Upon cooling to room temperature, the blue extract (Figure S1-B(i)) slowly turns pink, which is indicative of the exchange of NH_3_ ligands for water molecules or OH^–^ ions (Figure S1-B(ii)).^32^

**Figure 4:**
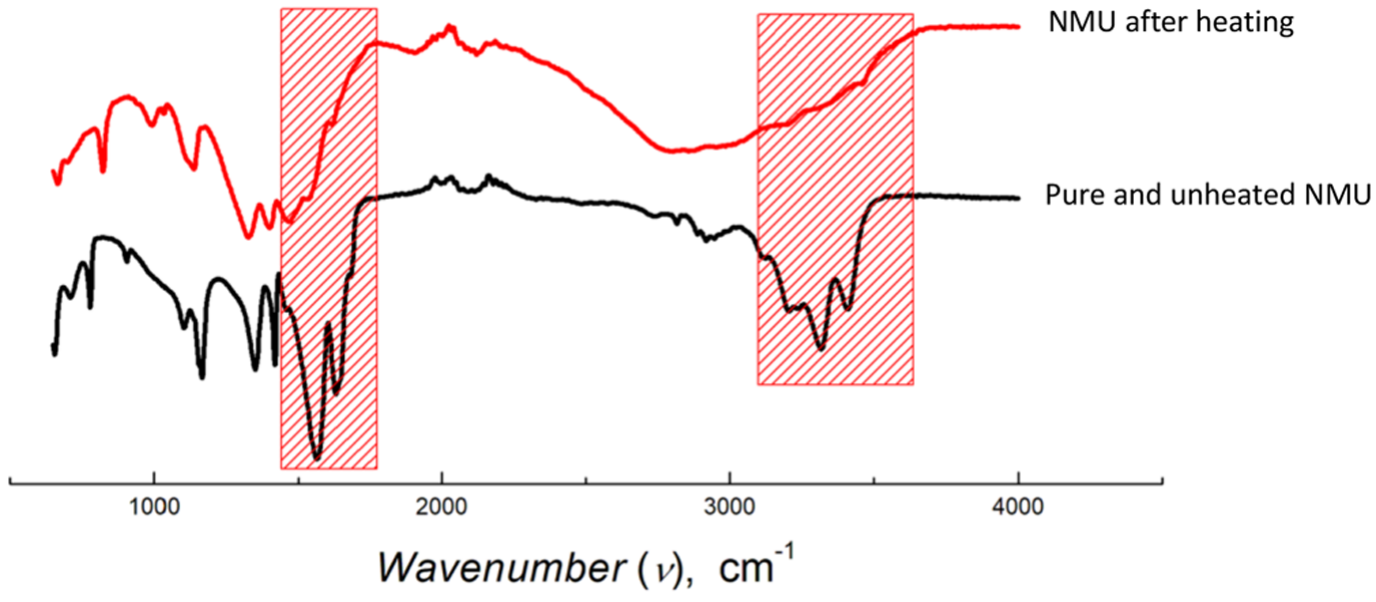
Infrared spectra of pure NMU and the NMU after heating containing the biuret derivative.

FT-IR spectra of the NMU after heating revealed the presence of the biuret derivative, as reflected in the diminished intensity of the primary amide bands compared to the pure and unheated NMU, supporting its consumption in the dimerization process (Figure 4). This reaction process and the associated blue color were also observed in the other NMU-containing ni-DESs, with the ni-DESs with higher NMU contents showing the most intense color (Figure 1).

**Figure 5:**
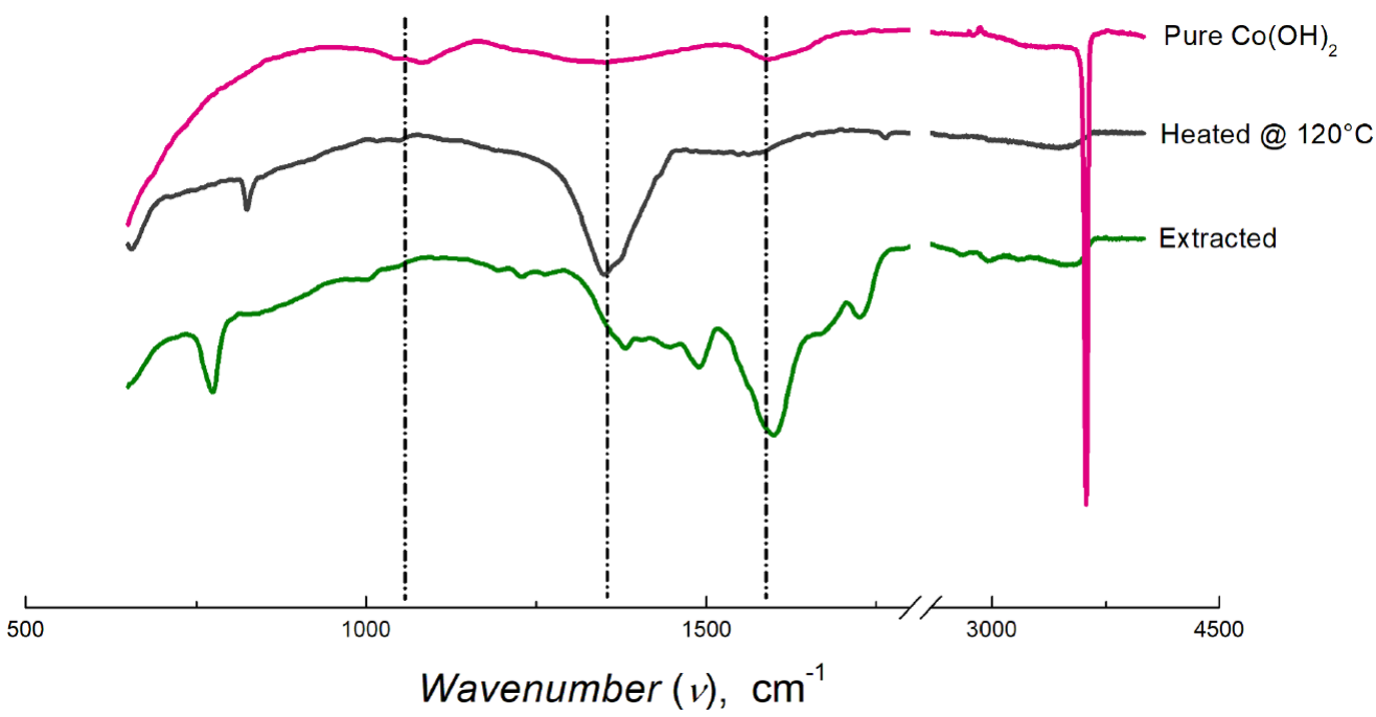
IR spectra of extracted, annealed (120 °C for 12 h), and calcinated (500 °C for 5 h) cobaltous powder extracted from LCO using the NMU-A ni-DES. For comparison, the IR spectrum of pure Co(OH)_2_ (β-form) is also given.

The blue-colored LCO NMU-A extract was treated with an excess of water (pH 6.4, see section 4.2.1). The resulting bluish-green precipitate was collected by centrifugation, and the pellet was repeatedly redispersed in water and recentrifuged twice before drying in an oven for 2 h at 120 °C. SEM images revealed the polydisperse nature of agglomerated recovered cobalt powder (Figure S2-A). To further investigate the nature of the recovered material, powder X-ray diffraction measurements were performed. These showed a broad pattern indicating the amorphous nature of the extracted cobalt powder (Figure S2-B1). After annealing at 120 °C for 12 h, the peaks were well resolved (Figure S2-B2), denoting the presence of oxyhydroxides of cobalt,^33^ as indicated from the peaks around 38.9, 50.6, 62, and 69.2 corresponding to the lattice faces of (012), (015), (107), and (113), respectively. X-ray photoelectron spectroscopy of the precipitated cobalt powder indicates the presence of Co with the peaks at 781.2, 782.6, and 786.6 upon deconvoluting the band at 781 eV (Figure S2-C). The 15.2 eV difference in the Co 2p_1/2_ and Co 2p_3/2_ spin states is typical for cobalt (not shown).^34^ The presence of cobalt hydroxide and lithium can be inferred from the peak at 786.6 eV,^35^ and the presence of lithium can also be inferred from the peak around 55 eV (Figure S2-D). IR spectra of the precipitated cobalt powder after annealing (Figure 5) show noticeable bands at 1367, 1639, and 3500 cm^–1^ for the Co–OH bond along with bending and stretching modes of the −OH group, respectively, which are attributed to the hydroxides and trapped water molecules. Importantly, IR spectral bands are similar to those of pure Co(OH)_2_ after annealing, and the absence of amide bands indicates that the ni-DES components (NMU and A) have been effectively removed.

**Figure 6:**
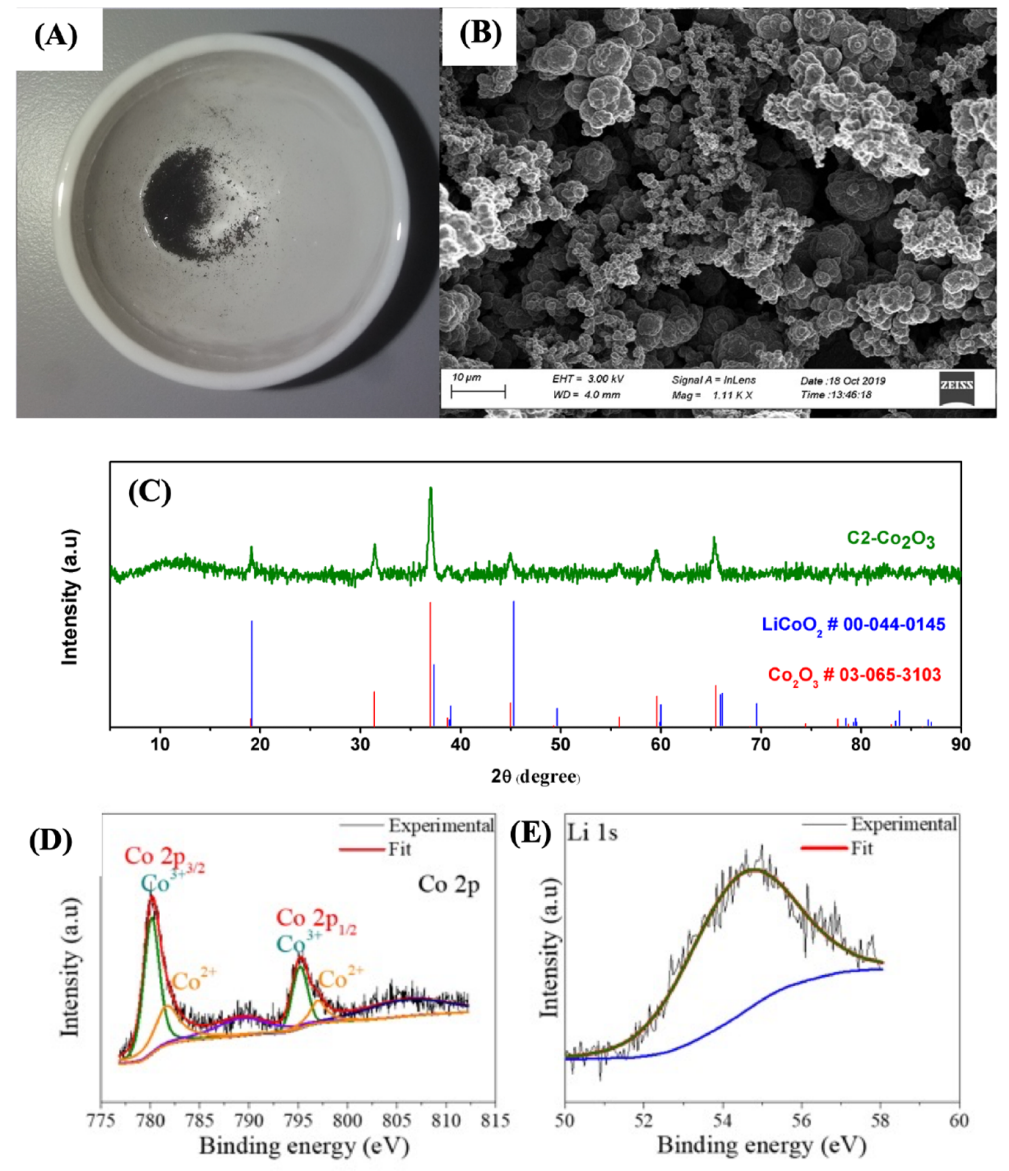
(A) Image, (B) electron micrograph, (C) powder XRD pattern, and (D and E) binding energy profiles (Co 2p and Li 1s, respectively) of recovered cobalt oxide.

The recovered cobalt powder was calcinated to convert it into a pure crystalline form. The bluish-green precipitate of the recovered cobalt turned black after calcination at 500 °C (Figure 6A). Figure 6B shows the SEM image of coarse particles of calcinated crystalline cobalt oxide (Co_2_O_3_), the identity of which was confirmed by XRD, XPS, and FT-IR. The powder XRD pattern of the calcinated cobalt extract (Figure 6C) closely resembles that of Co_2_O_3_ (JCPDS 03-065-3103). The peaks corresponding to hydroxide moieties seen in the extract prior to calcination, namely, those at 38.9°, 50.6°, 62°, and 69.2°, essentially disappear.

The binding energy profile obtained by XPS of the calcinated cobalt powder (Figure 6D and E) shows the Li 1s band at 54.6 eV and the Co 2p band around 781 eV, which upon deconvolution reveals peaks corresponding to Co_2_O_3_ at 780.1 and 781.2 eV.^36^ The virtual disappearance of the cobalt hydroxides band at 786.4 eV indicates the conversion of any amorphous oxyhydroxide and hydroxides of cobalt into more crystalline Co_2_O_3_. The XRD pattern reveals that cobalt oxide is the primary product, though the feasibility of LiCoO_2_ formation in the presence of lithium moieties cannot be ruled out. IR spectra of the calcined cobalt powder show metal–oxygen stretching and bending bands at 1430 and 831 cm^–1^, respectively^37^ (Figure 5). The octahedral form of Co^3+^ in Co_2_O_3_ can be inferred from the band at 531 cm^–1^.^38^ Again, the absence of bands for hydroxides (1639 and 3500 cm^–1^) confirms the formation of Co_2_O_3_ and conclusions drawn from the XPS studies. Based on the mass conversion determined from ICP-AES measurements (Table S5), nearly 85 ± 2% of the cobalt was recovered from the LCO as Co_2_O_3_.

**Figure 7:**
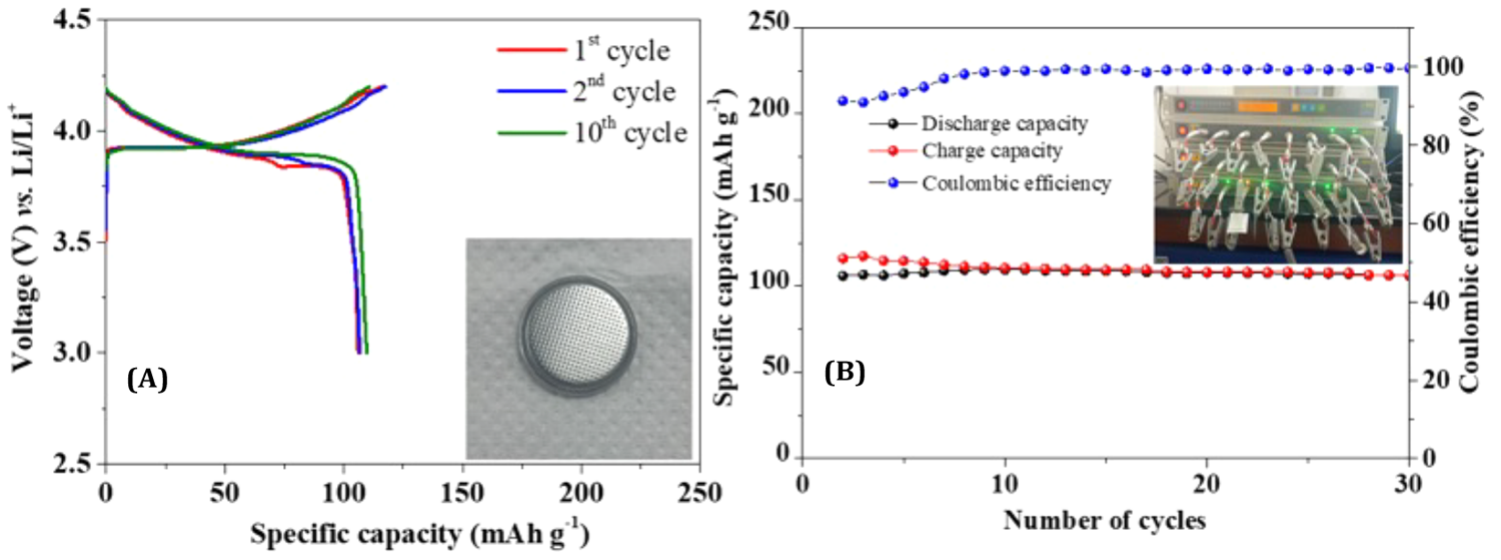
(A) Galvanostatic charge–discharge profile (inset shows the fabricated coin cell) and (B) cycling stability of a LCO-R coin cell at a 0.2 C rate (inset shows the coin cell testing).

Regenerated LCO (LCO-R) was synthesized from recovered cobalt oxide by mixing with a required amount of lithium hydroxide (0.15 mg of LiOH for 5 mg of LCO), as calculated from ICP-AES studies (Table S5), and calcinating the mixture at 500 °C for 6 h. A LCO-LiB fabricated using the LCO-R was tested for the charge–discharge cycles (see section 4.2.10). Figure 7A shows the galvanostatic charge–/discharge profile of the regenerated LCO-R cathode for the first, second, and tenth cycles performed at a 0.2 C rate. The discharge and charge plateaus are observed at 3.89 and 3.92 V vs Li/Li^+^, corresponding to the redox behavior of Co^3+^/Co^4+^.^39^ It delivers first-cycle discharge and charge capacities of 106 and 116 mAh g^–1^, respectively, and demonstrates the excellent reversible cycling performance of the LCO-R based LiB. In addition, the cell demonstrated a stable capacity of around 108 mAh g^–1^ for 30 cycles (Figure 7B) with a Coulombic efficiency of 99%, which is comparable with the performance of equivalent commercial LiBs.^40^

Subsequently, the discharged battery was stripped, and the cathode component was immersed in the NMP without further treatment (Figure S3-A). After sonicating the NMP for 10 min, the LCO layer, along with the conductive carbon matrix, is delaminated from the aluminum layer.

It combines with the liquid phase (Figure S3-B), while the aluminum foil can be removed mechanically (Figure S3-C). The PVDF adhesive layer easily detaches from the LCO particles and dissolves in the NMP, which can be removed in the subsequent centrifugation process. The pelleted LCO residue (Figure S3-D) was dried at 120 °C for 2 h, dispersed in NMU-A ni-DES, and heated to 180 °C for 24 h, while the appearance of dark blue hues in the extract confirms the extraction of cobalt (Figure S3-E). The ni-DES extract with cobalt ions was pipetted out and mixed with an excess of deionized water to precipitate the cobalt. The extraction efficiency of cobalt for this process was determined to be 79.66% from the ICP-AES measurement. The feasibility of ni-DES for its reactivity toward different parts of the cathode components of the spent LiBs during the extraction of cobalt has been verified separately. The components generally present in the battery, such as polyvinylidene fluoride (PVDF) binder, carbon black, and aluminum foil, were exposed to the ni-DES and were stable even after heating at 140 °C for 12 h (Figure S4-A–C). It was noted that the copper foil anode of the LiBs reacts to form a blue-colored extract when treated with the ni-DES at 100 °C, probably due to the dissolution of copper oxide in ammoniacal conditions^41^ (Figure S4-D).

Electrodeposition is a flexible and facile method for recovering cobalt metal from the extract in a thin-film format.^42^ A cobalt hydroxide thin-film was prepared using cobalt recovered from LCO extracts, as shown in Scheme 1. The LCO leachate in the NMU-A was treated with an excess of water, and the bluish-green precipitate thus obtained was washed repeatedly with water to remove the ni-DES components and dried at 120 °C in an oven for 2 h. Calculated quantities of the dried powder were dissolved in 1% acetic acid to convert the extracted cobalt into the corresponding acetate salt. Cobalt hydroxide was electrodeposited from this Co(OAc)_2_ solution on the gold surface with 0.1 M KNO_3_ as a supporting electrolyte (Figure S5-A). The bright green-colored coating, characterized with SEM, EDX, and IR spectroscopy (Figure S5), was cobalt in the hydroxide form.

In this study, the approach for cobalt extraction from LCO is based on the ripening effect followed by the leaching of metal moieties, which progress at higher temperatures and stringent basic conditions (nascent ammonia), respectively. The same strategy can be extended for the extraction of metals that react and form soluble complex under ammoniacal conditions. The present approach is more energy efficient, safe, and economical for Li and Co extraction than the pyrometallurgical,^43^ biometallurgical,^44^ and acid–base leaching methods^45^ (Table S6). Furthermore, the extraction efficiency (>90%) is comparable with those of other hydrometallurgical methods under milder conditions^18,19^ (Table S6).

## 3 Conclusions

In this study, we have demonstrated a novel and efficient method for cobalt extraction from spent LCO-based LiBs using a ni-DES comprised of *N*-methyl urea and acetamide. An extraction efficiency of >97% was obtained under optimized conditions. The efficiency of the reaction depended on the formation of a biuret derivative arising from the reaction of *N*-methyl urea. Recovered extracted cobalt was used to fabricate new fully functional new LiBs from which the cobalt could again be retrieved for subsequent reuse.

## 4 Methods

### 4.1 Chemicals

Lithium cobalt oxide (LCO, 99.9%), *N*-methyl urea (NMU, 97%), acetamide (A, 99%), urea (U, ACS reagent), *N*,*N*′-dimethylurea (NN′DMU, 99%), *N*-methylacetamide (NMA, 99%), *N*-methyl-2-pyrrolidone (NMP), potassium nitrate (ACS reagent), nitric acid, methanol (HPLC grade), and acetic acid (glacial) were all purchased from Sigma-Aldrich and used without any purification process unless otherwise mentioned. Gold-coated silicon wafers were purchased from Sigma-Aldrich. NMU was recrystallized from methanol. Ultrapure-grade water (resistivity 18.2 MΩ), purified using the Milli-Q gradient water filtration system (Millipore), was used for all purposes wherever necessary. Prior to usage, the Au thin films were immersed in a piranha solution (3:1, H_2_O_2_ (30%)/H_2_SO_4_ (conc.) *v*/*v*) for 30 s, washed with 5 mL of water five times (fresh water was replaced each time), dried flushed with N_2_ and immediately used for analysis.

### 4.2 Instrumentation and Related Protocols

#### 4.2.1 Extraction of Metals from LCO Using ni-DES

The NMU-A ni-DES was prepared by heating a known amount of acetamide at 80 °C under constant stirring at 500 rpm until it turned into a clear liquid. To that was added an equal amount of recrystallized *N*-methyl urea (*w*/*w*), and the mixture was heated at the same temperature until a homogeneous clear liquid phase was obtained. This liquid phase solidifies at a temperature of less than 43 °C. The ni-DESs were prepared freshly each time before the extraction process. The metal extraction experiments have been performed by mixing a known quantity of the LCO cathode material with 2 mL of NMU-A in a closed airtight vial (1.5 mm diameter, pyrex, Schott-Duran). The temperature dependence of the metal extraction process was monitored by placing the individual vials containing the mixture in an anodized aluminum heating block equipped with 1.6 mm diameter holes. The heating was continued over 50–180 °C for a specific time interval, and the leaching efficiency was calculated from eq 1. The effect of the LCO concentration on the leaching efficiency was evaluated by heating different amounts of LCO in NMU-A for 6, 12, 18, and 24 h at 180 °C. After each thermal treatment, the ni-DES leachate (with LCO particles) was carefully transferred to another glass vial (20 mL) using a Pasteur pipet and treated with an excess volume (five times the volume of leachate) of water (pH 6.4). The bluish green precipitate thus obtained was pelleted out by centrifugation at 4500 *g* for 10 min at 21 °C. then, the principate was washed again in water by redispersing it in the identical quantity of water used before, and the process was repeated twice. Finally, the precipitate was dried at 120 °C for 2 h in an oven and stored in a vacuum desiccator containing silica gel.

#### 4.2.2 Leaching Efficiency

The extraction efficiency of ni-DES was calculated using the following equation:
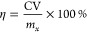
1where *C* is the concentration of the metal (in ppm) obtained from ICP-AES, *V* is the volume of leaching solution used (in L), and *m*_*x*_ is the initial mass of the desired metal (Li or Co) in the active material (in mg).^46^

#### 4.2.3 Inductively Coupled Plasma–atomic Emission Spectroscopy (ICP-AES)

The concentration of the desired metal in the leachate, after extraction with ni-DES under various conditions, was estimated using a PerkinElmer Avio 200 ICP-OES instrument. The extracted samples were diluted in 5 mL of 2% nitric acid. The flow rate of the gas nebulizer was set to 0.70 L/min. The wavelengths used in the axial mode to determine the cobalt and lithium concentration were 228.616, and 610.362 nm, respectively. The instrument was calibrated with five ICP standard solutions with a correlation coefficient not less than 0.999. The concentration of the extracted metals, expressed in ppm, are the mean values of at least three measurements, and the error bar shows the statistical dispersion (standard deviation) between the replicates.

#### 4.2.4 X-ray Diffraction (XRD) Measurement

Powder XRD patterns of the as extracted and calcinated powder were measured with a Bruker D8 Advance diffractometer using 40 kV and 40 mA Cu K_α_ radiation (λ = 1.54 Å). The powder sample was placed on a zero-background holder, and the measurements were performed at 0.5°/min incidence for 1.5 h at 300 K in the range of 10–85° (2θ range).

#### 4.2.5 Ultraviolet–visible Spectrometry

Absorption spectra of the ni-DES samples in the UV–visible range (700–250 nm) were recorded using UV-1800 spectrophotometer from Shimadzu Corporation (Tokyo, Japan). Baseline measurements in air were taken for pure NMU-A ni-DES at 100 °C in a 1 cm path length quartz cuvette and corrected for the measurements with samples. ni-DES treated with LCO at different temperatures between 120 and 180 °C were transferred into the quartz cuvette and were measured immediately within a span of 5 min.

#### 4.2.6 Fourier Transform–Infrared Spectroscopy (FT-IR)

Infrared spectra of the ni-DES and the extracted powder were recorded using an Agilent Cary 630 FTIR spectrometer (Agilent Technologies) provided with KBr optics and a complementary diamond attenuated total reflectance (ATR) sampling accessory. The samples were placed on a type IIa diamond crystal and measured in the ATR mode. Agilent MicroLab FTIR Software (Agilent Technologies) was used to collect a background spectrum and a sample spectrum and for further analysis. The samples were recorded within the 400–4000 cm^–1^ range with 32 scans and 4 cm^–1^ resolutions.

#### 4.2.7 X-ray Photoelectron Spectroscopy (XPS)

XPS measurements on the extracted and calcinated cobalt samples were carried out using a K-alpha X-ray photoelectron spectrometer (Thermo Fisher Scientific). The survey spectra were recorded with a 50.0 eV pass energy in the range from 0 to 1350 eV. The core-level spectra were recorded at a 200.0 eV pass energy and 0.1 eV increment. The pressure in the sample chamber was maintained at 5 × 10^–9^ mbar throughout the measurement. Binding energy data were collected for 20 scans, and the spot size of the incident beam was 400 μm. The resulting peaks were deconvoluted, and curve fitting was performed using XPS peakfit4.1 software.

#### 4.2.8 Scanning Electron Microscopy (SEM)

The topography of the recovered cobalt sample was mapped using a Leo 1550 Gemini instrument (Zeiss, Oberkochen, Germany) furnished with a field emission electron gun. Powdered samples and cobalt oxide thin-films, electrodeposited on Au-coated silicon wafers, were stuck on the double-sided carbon tape attached to alumina stubs. Before the SEM measurement, the samples were coated with a thin layer of platinum using a platinum sputtering unit (LEICA EM SCD 500) and inserted into the SEM instrument. A 3 kV potential was applied to the electron gun to generate the electron beam to scan the samples. The images recorded were presented without any further image processing.

#### 4.2.9 Metal Recovery

The extracted bluish-green precipitate of cobalt hydroxide was calcinated at 500 °C for 4 h to convert it into cobalt oxide and used for fabricating another lithium-ion battery (see section 4.2.10). Additionally, the cobalt was recovered in a thin-film format by the electrodeposition method. About 10 mg of the extracted cobalt hydroxide powder was dissolved in 500 μL of 1% acetic acid and converted to cobalt acetate. This solution was diluted with water, and a calculated quantity of KNO_3_ was added to yield 50 mM cobalt acetate and 0.1 M KNO_3_. Subsequently, this mixture was used as an electrolyte to deposit a cobalt hydroxide thin film under electrochemical conditions. Cyclic voltammetry conditions provided with a piranha-cleaned Au-coated silicon wafer (5 × 20 mm), platinum wire, and AgCl/Ag-KCl (saturated) as working, counter, and reference electrode, respectively, were used. The working electrode potential was scanned from 0 to 1.5 V for 10 cycles at a 50 mV/s scan rate. The bright green-colored coating on the working electrode was thoroughly washed with 5 mL of fresh water repeatedly three times.

#### 4.2.10 Lithium-Ion Battery Fabrication and Electrochemical Performance

The regenerated LiCoO_2_ (LCO-R), carbon black (Super C65), and polyvinylidene fluoride (PVDF) binder are mixed in an 8:1:1 ratio with *N*-methyl 2-pyrrolidone (NMP) solvent for 3 h to make an electrode slurry. The prepared slurry is coated on an aluminum foil current collector using a doctor blade and kept at 60 °C in an air oven for drying. After drying, the electrodes were cut into a circular disk with a diameter of 14 mm and used for cell fabrication with 1 M LiPF_6_ in EC/EMC (1:1) (ethylene carbonate/ethyl methyl carbonate) as an electrolyte. Celgard 2325 was used as a separator for this study. The CR 2032-coin cells were assembled in an argon-filled glovebox with moisture and oxygen levels less than 0.01 ppm. In this study, LCO-R is used as the working electrode, and a Li metal disk with a size of 14 mm is used as a reference and counter electrode. The electrochemical performance is measured in a half-cell configuration (LCO-R|electrolyte|Li). Galvanostatic charge/discharge studies are carried out at a 0.2 C rate in the potential window from 3 to 4.2 V vs Li/Li^+^.

#### 4.2.11 Peeling of Cathode Materials from Discharged LiBs

After repeated charge–discharge cycles, the assembled CR2032 coin cells were immersed in 10% KCl for 48 h to avoid self-ignition and short-circuiting (Scheme 1). The steel cover was peeled off to separate the cathode and anode components. The cathode layers coated on aluminum foil were cut into pieces and immersed in NMP, followed by sonication for 10 min to delaminate the aluminum layer. The residue collected after centrifugation was dried at 120 °C in a hot air oven for 2 h. The untreated LCO thus recovered was dispersed in NMU-A ni-DES, and the cobalt extraction process was carried out as mentioned in section. 4.2.1.

## Supporting Information Available

Additional information regarding the efficiency of extraction, characterization of the cobalt compounds, and recycling details (PDF)
