# Supplementary material for: Highly Efficient Recovery and Recycling of Cobalt from Spent Lithium-Ion Batteries Using an N-Methylurea–Acetamide Nonionic Deep Eutectic Solvent
Source: ACS Omega. 2023 Feb 13;8(7):6959–67. doi: 10.1021/acsomega.2c07780 (PMC9948188; doi:10.1021/acsomega.2c07780)
Supplement: Supplementary file 1 — ao2c07780_si_001.pdf [file ao2c07780_si_001.pdf]

Highly efficient recovery and recycling of cobalt from spent lithium-ion batteries using an *N*-methylurea–acetamide non-ionic deep eutectic solvent

Subramanian Suriyanarayanan [a], Mohana Priya Babu. [b], Raja Murugan [b], Divyamahalakshmi Muthuraj [b], Kothandaraman Ramanujam [b]* and Ian A. Nicholls [a]**

[a] Bioorganic & Biophysical Chemistry Laboratory, Linnaeus Centre for Biomaterials Chemistry, Department of Chemistry & Biomedical Sciences, Linnaeus University, SE-39182 Kalmar, Sweden

[b] Clean Energy Laboratory, Department of Chemistry, Indian Institute of Technology Madras, Chennai – 600 036, India

Correspondence:

subramanian.suriyanarayanan@lnu.se

rkraman@iitm.ac.in

ian.nicholls@lnu.se

Table of Contents

Figures and Tables

Figure S1 3

Figure S2 4

Figure S3 5

Figure S4 6

Figure S5 7

Table S1 8

Table S2 9

Table S3 10

Table S4 11

Table S5 12

(A)

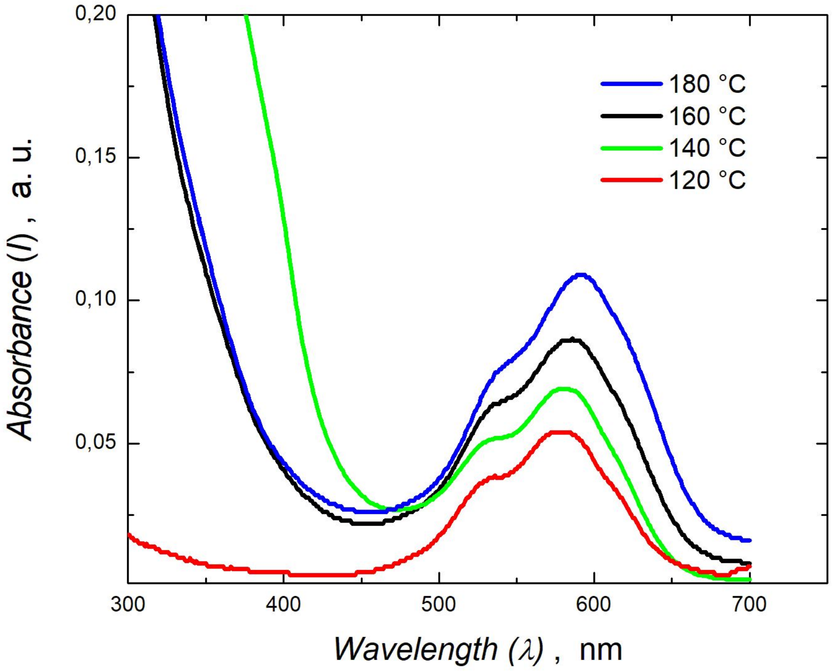

(B)

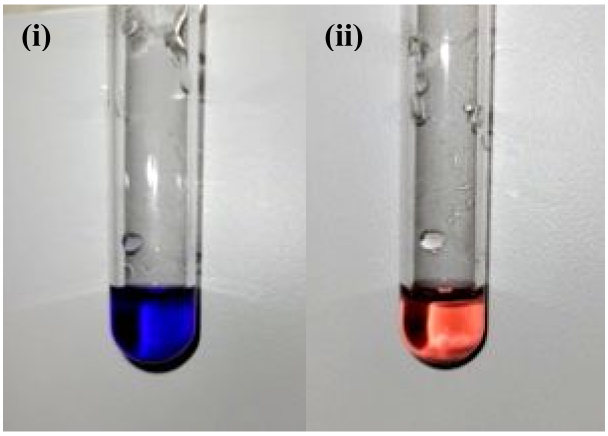

Figure S1. (A) UV spectra of the extracted cobalt complex upon heating LiCoO2 in NMU-A at different temperatures. (B) The ni-DES leachate containing LCO was observed (i) after heating at 180°C for 24 h and (ii) after cooling the same to 50°C.

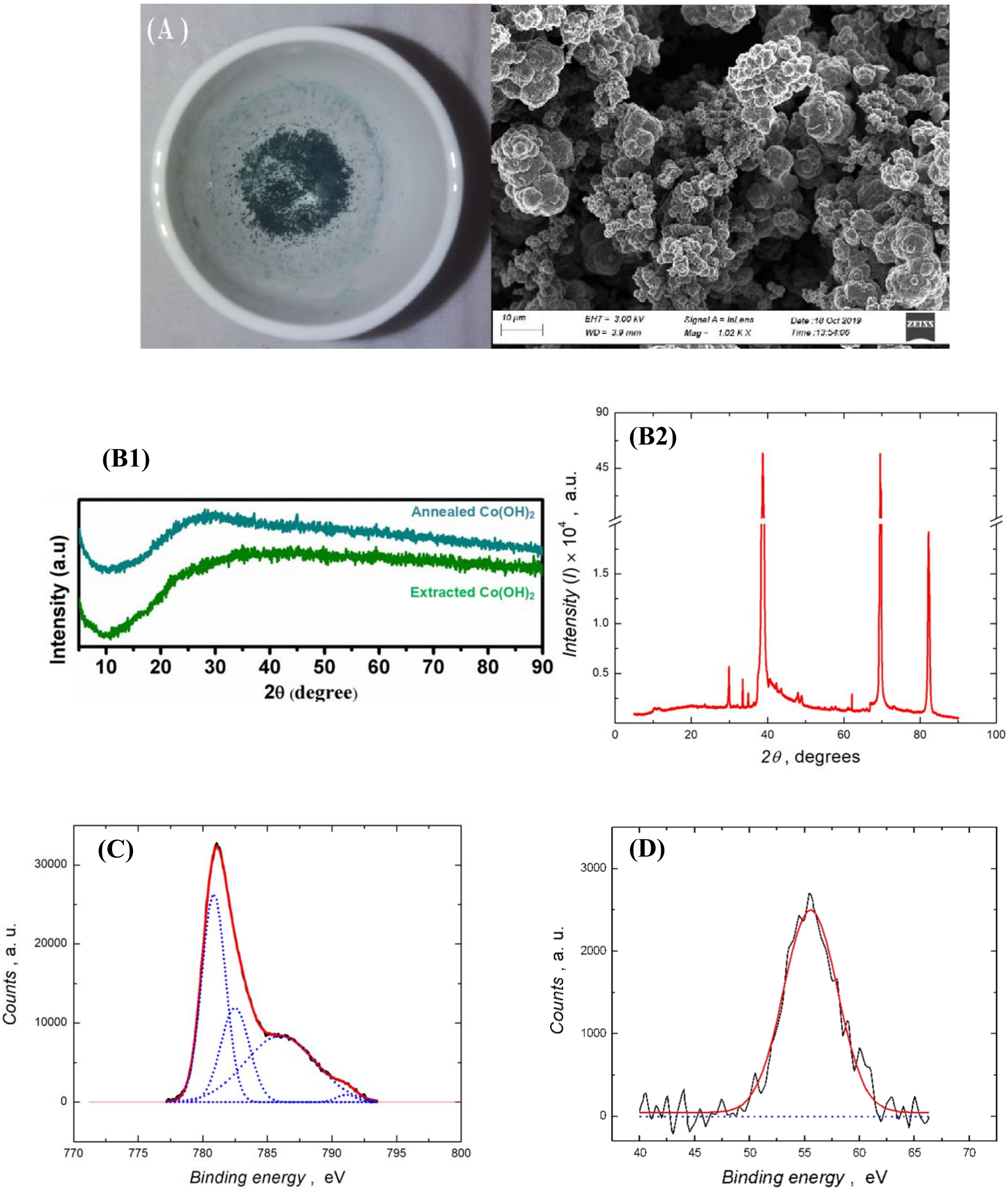

Figure S2. (A) SEM Image of the recovered cobalt powder (after rinsing with excess deionized water and drying overnight at 120˚C) from ni-DESs leachate containing cobalt ions extracted from $LiCoO_2$. (B1) Powder X-ray diffraction pattern of extracted, annealed cobalt extract and (B2) after annealing the same for 12 hours. C and D are the binding energy profile of the 12 h-annealed cobalt extract showing the Co2$p_{3/2}$ and Li1S peaks, respectively.

(A) (B) (C) (D) (E)

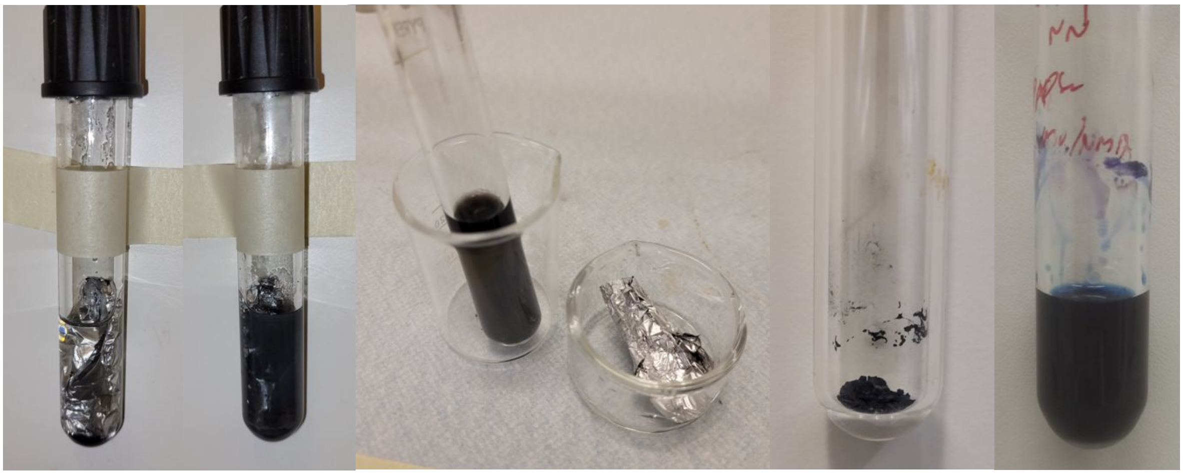

Figure S3. Extraction of cobalt from fabricated and discharged LiBs. (A) Immersion of cathode component (LCO coated aluminium foil) in NMP (B) sonication for 10 mins, (C) removal of aluminium layer (D) LCO pelleted residue (centrifugation @ 4500g for 10 mins) dried at 100 °C in a hot air oven for 2 hrs and (E) extraction of cobalt from LCO residue with NMU-A ni-DES

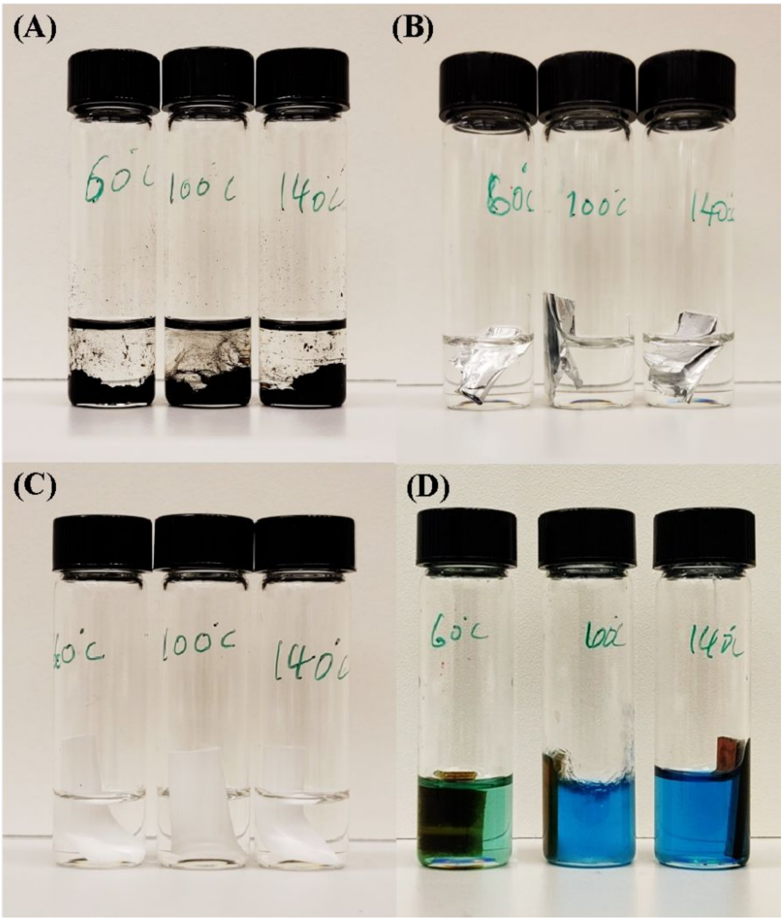

Figure S4. Reactivity of ni-DES with various other Li-ion battery components upon heating 2 g of NMU-A eutectic mixture at 60, 100 and 140°C containing (A) carbon black, (B) aluminium foil, (C) PVDF membrane and (D) copper foil.

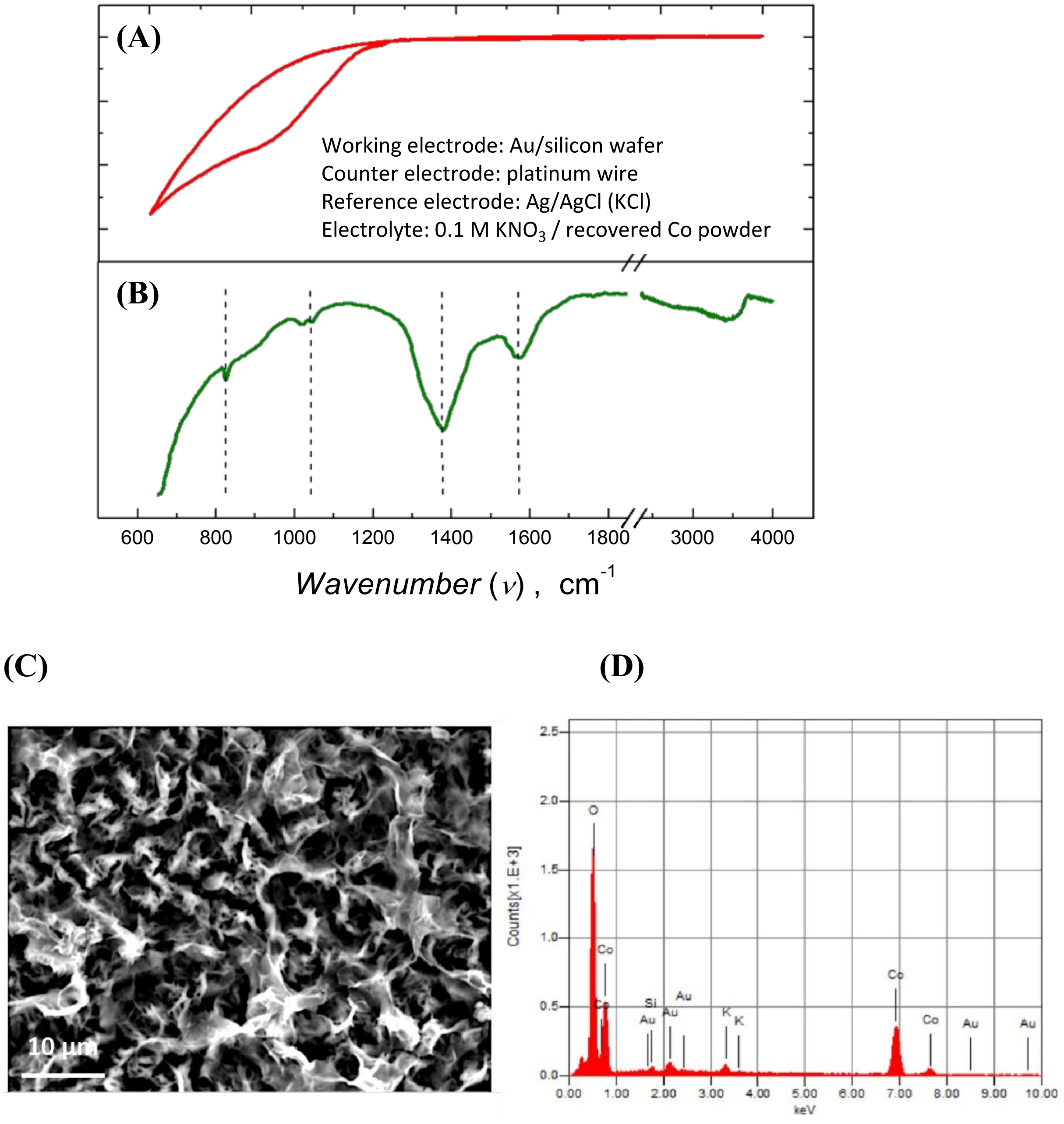

Figure S5. (A) Cyclic voltammogram for synthesizing of $Co(OH)_2$ thin film from the recovered cobalt precipitate from spent LiBs. (B) – (D) is the IR spectra, SEM image and EDX profile of the electro-synthesized $Co(OH)_2$ film.

Table S1. List of non-ionic deep eutectic solvents (ni-DESs) used to extract of cobalt and lithium.

ni-DES system [a]	Composition, wt %	Eutectic point, °C
NMA–A	70 : 30	15 ± 4
NMU–A	50 : 50	42 ± 3
NMA–NMU	80 : 20	14 ± 2
NMU–NN'DMU	50 : 50	49 ± 3
A–NN'DMU	50 : 50	43 ± 3
NMA–NN'DMU	70 : 30	15 ± 4

[a] A = acetamide, U = urea, NMU = *N*-methylurea, NN'DMU = *N*,*N*′-dimethylurea, and NMA = *N*-methylacetamide.

Table S2. Effect of temperature on the extraction of cobalt and lithium from LCO in ni-DES. Concentrations of Cobalt and extraction efficiency are calculated for 20 mg LCO in 2 mL of the ni-DES, heated at the respective temperature for 24 h. Metal concentration was averaged out and the standard deviation for three different measurements at each temperature.

Extracted metal	Temperature, T (°C)	Average concentration, c (ppm) × 10^2	Standard deviation × 10^2	Extraction efficiency, η (%)
Co	60	0.0029	0.0023	<0.01
	80	0.043	0.013	0.07
	100	0.23	0.063	0.37
	120	8.6	2.5	14
	140	12	1.3	20
	160	29	2.4	49
	180	54	3.9	91
Li	60	0.0012	0.0011	0.01
	80	0.0009	0.0015	0.01
	100	0.0035	0.0051	0.05
	120	1.1	0.23	15
	140	1.4	0.20	21
	160	3.3	0.48	47
	180	6.4	1.0	91

Table S3. Time-dependent cobalt extraction from LCO in ni-DES. Concentrations of Cobalt and extraction efficiency are calculated for 20 mg LCO in 2 mL of the ni-DES, heated at the respective temperature for a specified time duration. Cobalt concentration was averaged out and the standard deviation for three different measurements at each temperature.

Temperature, T (°C)	Time, t (h)	Average Cobalt concentration, c (ppm) × 10^2	Standard deviation × 10^2	Extraction efficiency, η (%)
100	12	0.19	0.050	0.31
	24	0.23	0.063	0.37
	36	0.6	0.18	0.99
	48	1.7	0.36	2.9
120	12	5.7	1.5	9.6
	24	8.6	2.5	14
	36	9.0	2.8	15
	48	11	2.0	19
140	12	6.8	2.1	11
	24	12	1.3	20
	36	15	1.3	25
	48	15	1.8	26
160	12	27	4.2	46
	24	29	2.4	49
	36	33	2.2	55
	48	35	5.0	58
180	12	49	5.4	82
	24	54	3.9	91
	36	57	2.1	95
	48	59	2.0	98

Table S4. Extraction of metals from $LiCoO_2$ (LCO) in ni-DES, with various concentration of LCO powder, initially added and mixed with a finite amount (2 mL) of ni-DES and heated at 180°C. Cobalt concentration was averaged out and the standard deviation for three different measurements at each temperature.

Initially added LCO, *c* (mg/mL)	Solid to liquid ratio, g/L	Time, *t* (h)	Cobalt concentration, *c* (ppm) × 10^2	Standard deviation × 10^2	Extraction efficiency, *η* (%)
1	0.5	6	0.50	0.086	17
		12	0.84	0.10	28
		18	1.1	0.15	38
		24	1.5	0.18	52
2	1	6	1.7	0.20	28
		12	2.2	0.24	37
		18	3.0	0.17	50
		24	3.7	0.16	62
5	2.5	6	5.2	0.64	35
		12	7.4	0.62	49
		18	10	0.45	69
		24	13	0.54	88
10	5	6	15	1.7	49
		12	22	1.6	73
		18	24	0.98	80
		24	27	1.0	89
20	10	6	40	4.5	67
		12	49	4.4	82
		18	51	2.0	85
		24	54	3.9	91
50	25	6	80	7.3	53
		12	100	9.1	69
		18	120	9.9	79
		24	130	11	86
100	50	18	230	-	75
		24	250	-	82

Table S5. ICP-AES analysis of calcinated cobalt powder (5 mg dissolved in 10 mL of 2% HNO_3) obtained from the extraction with LCO using NMU-A ni-DES (See table 3-SI).

Element symbol and Wavelength (nm)	Concentration, ppm × 10^2
Co 228.616	3.0
Li 670.784	0.34

Table S6. List of other reported methods for the leaching of LCO from spent LiBs for Li and Co recovery.

Processing method	Leaching agent	Process conditions	Post treatment	Efficiency of extraction	Refs.
Vacuum pyrolysis	-	600 °C / 30 mins	Acid (H_2SO_4)	99 % Li and 99 % Co	[1]
Chlorination roasting	Cl_2 gas	900 °C / 90 mins	-	99 % Li and 99 % Co	[2]
Inorganic acid leaching	$H_2SO_4 + H_2O_2$	90 °C /150 mins	Acid (H_2SO_4)	98 % Li and 99 % Co	[3]
Organic acid leaching	CH_3SO_3H / H_2O_2	70 °C / 60 mins	-	99 % Li and 99 % Co	[4]
Bio-leaching	S. thermosulfidooxidans	40 °C / 48 h	-	98.1 % Li and 96.3 % Co	[5]
Hydrometallurgy	Choline chloride + ethyleneglycol	220 °C / 24 h	Base (Na_2CO_3)	> 90 % Li and > 90 %ß Co	[6]
Hydrometallurgy	*N*-methylurea + acetamide	180 °C / 24 h	Water	90.91 % Li and 90.78 % Co	This work

REFERENCE

(1) Sun, L.; Qiu, K. Vacuum pyrolysis and hydrometallurgical process for the recovery of valuable metals from spent lithium-ion batteries. *Journal of Hazardous Materials* **2011**, *194*, 378-384. DOI: https://doi.org/10.1016/j.jhazmat.2011.07.114.

(2) Barrios, O. C.; González, Y. C.; Barbosa, L. I.; Orosco, P. Chlorination roasting of the cathode material contained in spent lithium-ion batteries to recover lithium, manganese, nickel and cobalt. *Minerals Engineering* **2022**, *176*, 107321. DOI: https://doi.org/10.1016/j.mineng.2021.107321.

(3) Chu, W.; Zhang, Y.; Chen, X.; Huang, Y.; Cui, H.; Wang, M.; Wang, J. Synthesis of LiNi0.6Co0.2Mn0.2O2 from mixed cathode materials of spent lithium-ion batteries. *Journal of Power Sources* **2020**, *449*, 227567. DOI: https://doi.org/10.1016/j.jpowsour.2019.227567.

(4) Wang, B.; Lin, X.-Y.; Tang, Y.; Wang, Q.; Leung, M. K. H.; Lu, X.-Y. Recycling LiCoO2 with methanesulfonic acid for regeneration of lithium-ion battery electrode materials. *Journal of Power Sources* **2019**, *436*, 226828. DOI: https://doi.org/10.1016/j.jpowsour.2019.226828.

(5) Liu, X.; Liu, H.; Wu, W.; Zhang, X.; Gu, T.; Zhu, M.; Tan, W. Oxidative Stress Induced by Metal Ions in Bioleaching of LiCoO2 by an Acidophilic Microbial Consortium. *Frontiers*

in Microbiology **2020**, *10*, 3058, Original Research. DOI:
https://doi.org/10.3389/fmicb.2019.03058.

(6) Tran, M. K.; Rodrigues, M.-T. F.; Kato, K.; Babu, G.; Ajayan, P. M. Deep eutectic solvents for cathode recycling of Li-ion batteries. *Nature Energy* **2019**, *4* (4), 339-345. DOI: 10.1038/s41560-019-0368-4.
